# Oral Melanoma: A South American Collaborative Series of 21 Cases

**DOI:** 10.1007/s12105-026-01936-w

**Published:** 2026-06-17

**Authors:** José Alcides Almeida de Arruda, Mariana Villarroel-Dorrego, Claudia Patricia Peña-Vega, Juan Pablo Rodríguez-Mora, Gerardo Gilligan, René Panico, Javier Gimenez, Verónica Flück, Ángeles Castrillo, Georgina Scalise, Laura Leguina, Mariene da Silva Monteiro, Gerhilde Callou Sampaio, Victor Zanetti Drumond, Jefferson R. Tenório, Bruno Augusto Benevenuto de Andrade

**Affiliations:** 1 Department of Oral Diagnosis and Pathology, School of Dentistry, Universidade Federal do Rio de Janeiro, R. Rodolpho Paulo Rocco, n. 325, 1st floor, Cidade Universitária, Rio de Janeiro, RJ CEP: 21.941-902 Brazil; 2 School of Dentistry, Universidad Central de Venezuela, Caracas, Venezuela; 3 Department of Basic Science and Oral Medicine, School of Dentistry, Universidad Nacional de Colombia, Bogotá, Colombia; 4 Department of Oral Medicine, School of Dentistry, Universidad Nacional de Córdoba, Córdoba, Argentina; 5 Servicio de Estomatología Oncológica, Hospital de Oncología Marie Curie, Buenos Aires, Argentina; 6 Servicio de Anatomía Patológica, Hospital de Oncología Marie Curie, Buenos Aires, Argentina; 7 Department of Oral Surgery, Pathology and Clinical Dentistry, School of Dentistry, Universidade Federal de Minas Gerais, Belo Horizonte, Brazil; 8 Department of Oral and Maxillofacial Surgery and Pathology, School of Dentistry, Universidade de Pernambuco, Recife, Brazil

**Keywords:** Head and neck neoplasms, Malignant melanoma, Melanoma, Oral cavity, Oncology, Pigmentation

## Abstract

**Background:**

Oral melanoma is a rare and aggressive malignant neoplasm that typically exhibits variable degrees of pigmentation. Amelanotic variants further complicate clinical diagnosis by mimicking non-neoplastic lesions. The present study aimed to describe the clinicopathological characteristics, management, and outcomes of oral melanoma in South America.

**Methods:**

This collaborative retrospective study included cases diagnosed between 2003 and 2025 from five oral pathology and oral medicine centers in Argentina, Venezuela, Colombia, and Brazil. Clinicopathological, treatment, and follow-up data were analyzed descriptively.

**Results:**

Twenty-one cases of primary oral melanoma were analyzed. The mean age was 63.0 years, with a slight female predominance (52.4%). Most lesions were asymptomatic at diagnosis (78.3%) and involved the palate/maxillary gingiva (66.7%). Clinically, tumors (37.9%) and macules (27.6%) were the most frequent presentations. Histologically, melanotic melanoma predominated (76.2%). Melan-A, HMB-45, SOX10, and S-100 protein were the most frequently used immunohistochemical markers. Surgical resection was performed in 85.7% of cases. During follow-up, 52.6% of patients died.

**Conclusion:**

Data corroborate the clinicopathological profile of oral melanoma and expand current knowledge by documenting novel South American cases, underscoring its marked clinical heterogeneity, poor prognosis, and the critical importance of early recognition.

**Supplementary Information:**

The online version contains supplementary material available at 10.1007/s12105-026-01936-w.

## Introduction

Melanoma is a malignant neoplasm of melanocytic origin. Mucosal melanoma comprises less than 1% of all melanomas and arises from melanocytes within the mucous membranes of the head and neck, anorectal, and genitourinary tracts, with the head and neck region accounting for approximately 50–55% of cases [1–3]. Within this subgroup, oral melanoma is exceptionally rare, representing 0.2–0.8% of all melanomas and 0.5–0.9% of all malignancies [2–6]. Recent evidence indicates an increasing incidence of melanoma accompanied by declining mortality rates in high-income countries [7].

Clinically, the palate and maxillary gingiva are the most frequently affected sites. Individuals are often asymptomatic; consequently, diagnosis commonly occurs when lesions are already advanced [4, 6, 8, 9]. Accordingly, oral melanoma is characterized by an unfavorable prognosis, with overall survival rates of 34% at five years and 15.4% at 10 years [6, 10]. Predictors of poorer survival include tumor size, lymph node metastasis, and non-palatal location [10]. Oral melanomas lack established environmental risk factors, occur independently of ultraviolet radiation exposure, and exhibit a molecular profile distinct from that of cutaneous melanomas [2, 11]. Surgical resection, with or without adjuvant radiotherapy, is associated with improved survival outcomes compared with non-surgical approaches [12]. Additionally, oral amelanotic melanoma is ultrarare, with fewer than 100 cases published in the literature. Its clinical diagnosis is particularly challenging because it may emulate non-neoplastic lesions (e.g., pyogenic granuloma, peripheral giant-cell granuloma, and fibrous hyperplasia), thereby contributing to diagnostic delays and/or misdiagnosis [13–15].

Epidemiological data indicate that the United States, Japan, Italy, and India report the highest numbers of oral melanoma cases [6, 16]. In Japan, oral melanoma accounts for 7.5% of all malignant melanomas and 34.4% of mucosal melanomas [2]. Conversely, epidemiological information from South America remains scarce compared with that from high-income countries [3, 5, 16–21]. In Brazil, for example, records from 2000 to 2016 showed that 23.1% of melanoma cases involved the lips, oral cavity, and pharynx [21]. In this context, detailed clinicopathological characterization of oral melanoma through a collaborative South American effort is important for delineating its diagnostic spectrum and informing evidence-based management [2]. The aim of the present study was to describe cases of oral melanoma diagnosed in Argentina, Venezuela, Colombia, and Brazil, offering an overview of demographic and clinicopathological features, treatment, and outcomes.

## Materials and Methods

### Study Design and Ethical Aspects

This retrospective multicenter series included 21 oral melanoma cases diagnosed between 2003 and 2025, with records retrieved from the archives of oral pathology and oral medicine centers in South America. The study was reported in accordance with the Strengthening the Reporting of Observational Studies in Epidemiology (STROBE) checklist [22]. Ethics approval was granted by the Research Ethics Committees (No. 26102024 and No. 29651420500005257), and Material Transfer Agreements were drafted and signed to formalize the collaborative framework. Participant anonymity and data confidentiality were maintained in accordance with the Declaration of Helsinki.

### Participating Centers

Participating centers included the Servicio de Estomatología Oncológica and Servicio de Anatomía Patológica, Hospital de Oncología Marie Curie, Buenos Aires, Argentina (*n* = 8); the Department of Oral Medicine, Facultad de Odontología, Universidad Nacional de Córdoba, Córdoba, Argentina (*n* = 3); the School of Dentistry, Universidad Central de Venezuela, Caracas, Venezuela (*n* = 7); the Department of Basic Science and Oral Medicine, School of Dentistry, Universidad Nacional de Colombia, Bogotá, Colombia (*n* = 2); and the Service of Oral Medicine, School of Dentistry, Universidade Federal do Rio de Janeiro, Rio de Janeiro, Brazil (*n* = 1).

### Diagnostic Rendering

Archived formalin-fixed, paraffin-embedded tissue blocks were retrieved, and 4-µm-thick sections were prepared and stained with hematoxylin and eosin for histopathological evaluation. The diagnosis of oral melanoma was established based on conventional histomorphological criteria and was characterized by malignant tumor cells exhibiting polymorphic growth patterns, including spindle-shaped, epithelioid, plasmacytoid, rhabdoid, round-cell, or clear-cell morphology. Marked cytologic atypia and/or undifferentiated morphologic features were required for inclusion. The presence of melanin pigment was not mandatory for diagnostic rendering, since amelanotic melanomas may show absent or only minimal pigmentation. In pigmented tumors, melanin deposition served only as an ancillary morphologic clue supporting melanocytic differentiation, whereas in amelanotic tumors, the diagnosis was rendered based primarily on immunohistochemical evidence of melanocytic lineage, interpreted together with the morphologic and clinicopathologic context. Diagnostic criteria were applied in accordance with the definition of mucosal melanoma in the 5th edition of the World Health Organization (WHO) Classification of Head and Neck Tumors [23]. Immunohistochemical data were reviewed to support diagnostic confirmation. Metastatic melanomas involving the oral cavity and cases with insufficient clinicopathological documentation were excluded.

### Data Collection

Collected variables included sex, age at diagnosis, skin color (White and non-White/Black), anatomical location of the lesion, clinical presentation (unifocal or multifocal), symptomatology at diagnosis, lesion size (measured as the greatest clinical dimension, in centimeters), clinical diagnostic hypothesis, histological type (melanotic or amelanotic), immunohistochemical markers, treatment modalities, follow-up duration (in months), and patient outcome (dead or alive).

### Data Analysis

Data were tabulated using Microsoft Office Excel (Microsoft, Redmond, WA, USA) and subjected to descriptive statistical analysis. Categorical variables were summarized as absolute frequencies and percentages using GraphPad Prism version 8.0.0 for Windows (GraphPad Software, San Diego, CA, USA).

## Results

### Demographic Characteristics

**Fig. 1:**
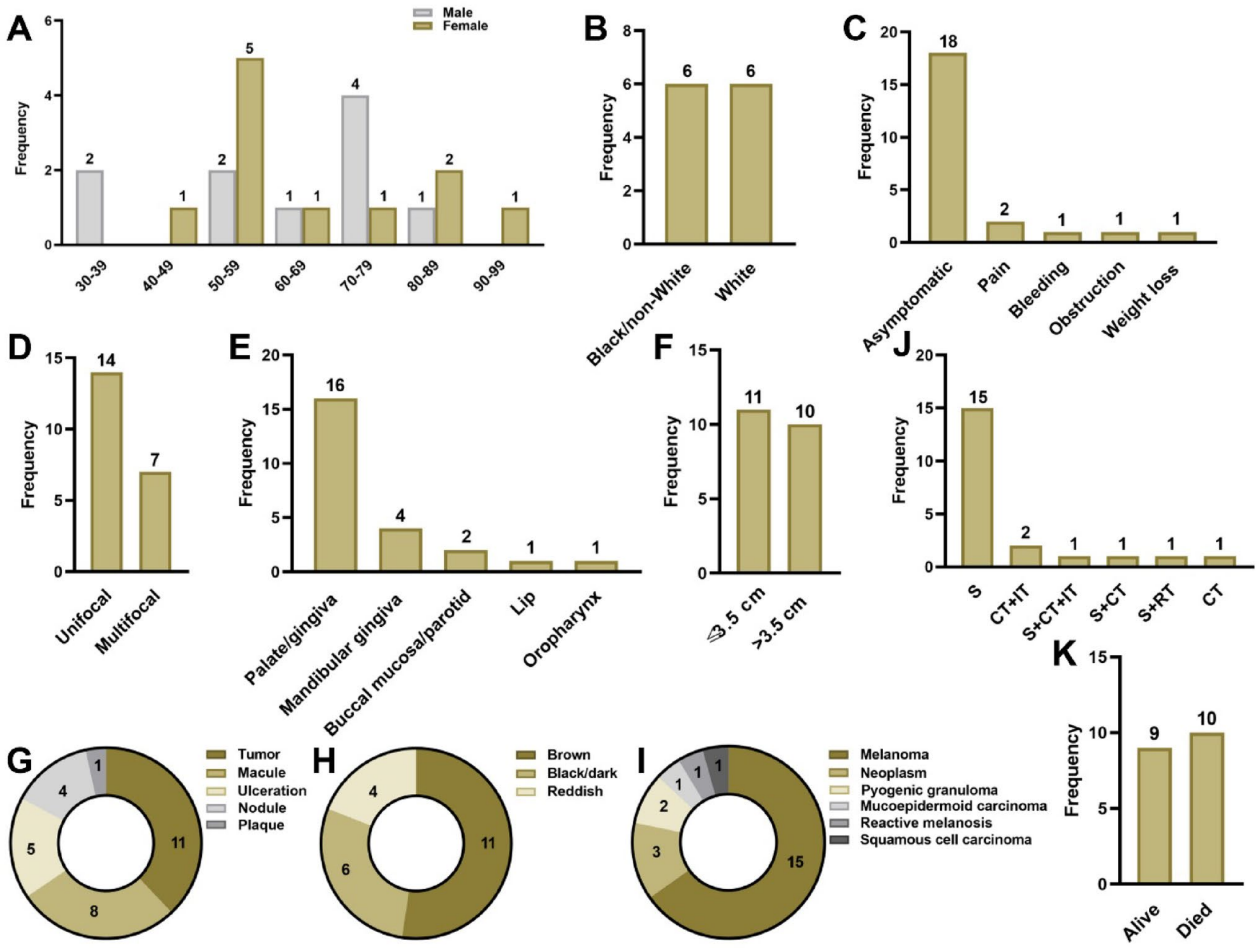
Clinicodemographic, treatment, and outcome data of oral melanoma cases. **A** Age distribution according to sex. **B** Self-reported skin color. **C** Symptomatology. **D** Number of affected oral sites. **E** Anatomical distribution of lesions. **F** Lesion size. **G** Clinical morphology of the lesions. **H** Lesion color. **I** Clinical diagnostic hypothesis. **J** Treatment modalities (CT, chemotherapy; IT, immunotherapy; RT, radiotherapy; S, surgery). **K** Patient outcomes. Note: the unit of analysis for symptomatology, anatomical location, lesion morphology, and clinical diagnostic hypothesis was not the number of individuals, as more than one feature could occur in the same case

A total of 21 oral melanoma cases were included, of which 10 (47.6%) were from Argentina, eight (38.1%) from Venezuela, two (9.5%) from Colombia, and one (4.8%) from Brazil. The mean age at diagnosis was 63.0 ± 16.2 years (median: 60; range: 30–90). Eleven (52.4%) patients were women and 10 (47.6%) were men, corresponding to a female-to-male ratio of 1.1:1. The mean age among women was 64.7 ± 15.6 years (median: 56; range: 49–90), whereas the mean age among men was 61.1 ± 16.6 years (median: 66; range: 30–80). The most frequently affected age group was 50–59 years (*n* = 7; 33.3%) (Fig. 1A). Information on skin color was available for 12 individuals, with an equal distribution between White and Black/non-White patients (*n* = 6; 50% each) (Fig. 1B). Detailed clinicodemographic information for all cases is provided in Supplementary Table 1.

### Clinical Aspects

**Fig. 2:**
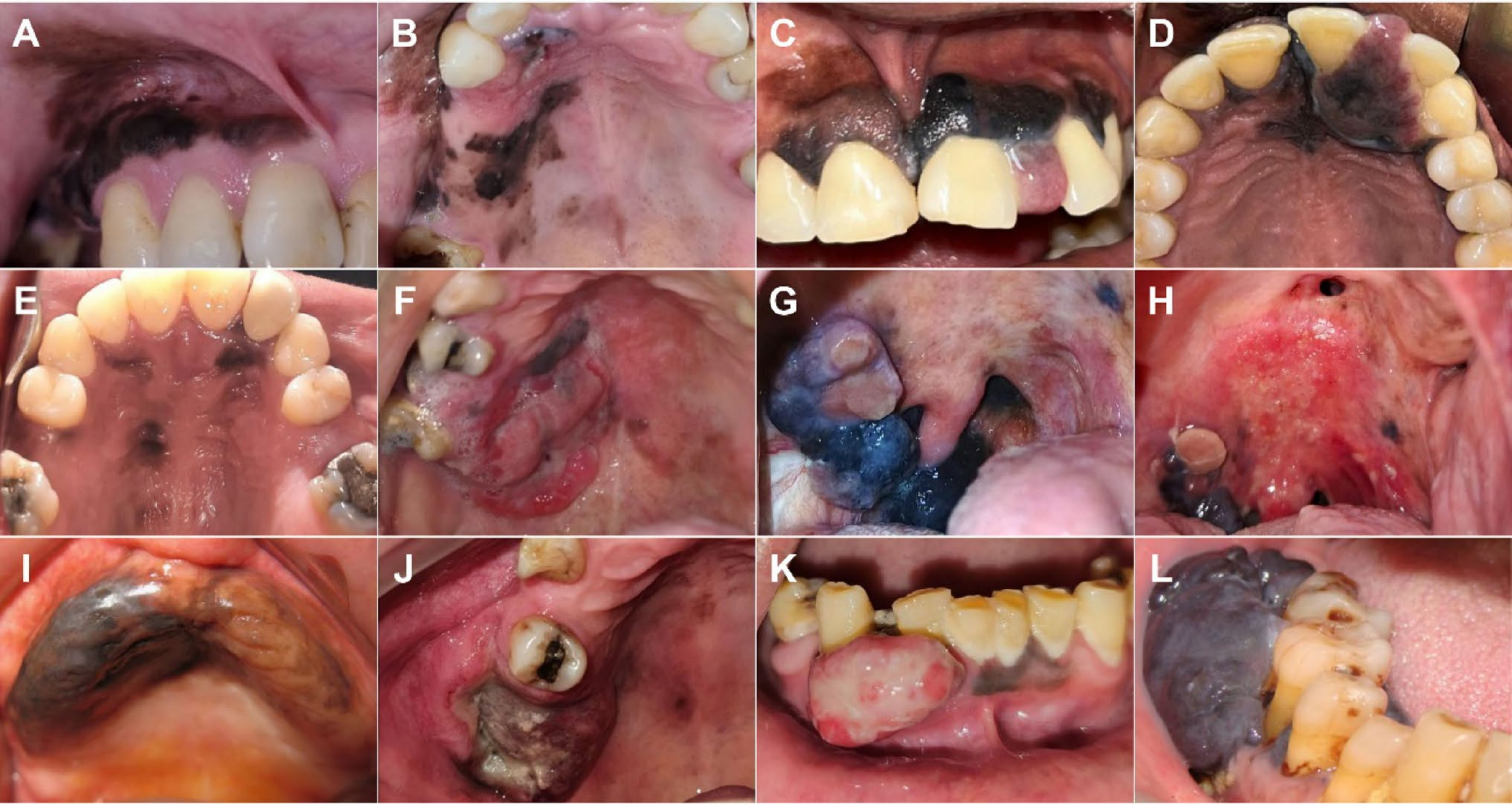
Clinical presentations of oral melanotic melanoma cases. **A**, **B** Contiguous brownish, irregular macules involving the maxillary gingiva, vestibule, and hard palate. **C**, **D** Contiguous black-brown macules involving the anterior maxillary gingiva, vestibule, and hard palate. Note a reddish nodule on the palate and interdental gingiva with areas of black pigmentation. **E** Multifocal brownish macules on the hard palate. **F** Ulcerated exophytic tumor on the posterior hard palate contiguous to the maxillary tuberosity, with reddish and blackened areas. **G** Exuberant black-reddish tumor involving the soft palate, tonsil, and oropharynx. **H** The same patient developed superimposed herpetic lesions during radiotherapy. Note the ulcerated, crusted yellowish and reddish areas involving the soft palate, uvula, and tonsillar pillar. **I** Blackish macule involving the entire edentulous hard palate. **J** Ulcerated exophytic tumor of the maxillary tuberosity. Note multifocal brownish macules on the hard and soft palate. **K** Yellowish-reddish oval nodule of the mandibular gingiva. Note an adjacent brownish macule on the attached gingiva of the incisors. **L** Irregular brownish-black tumor involving the right posterior mandibular gingiva of the molar region and vestibule. Note that the lingual aspect is also involved

**Fig. 3:**
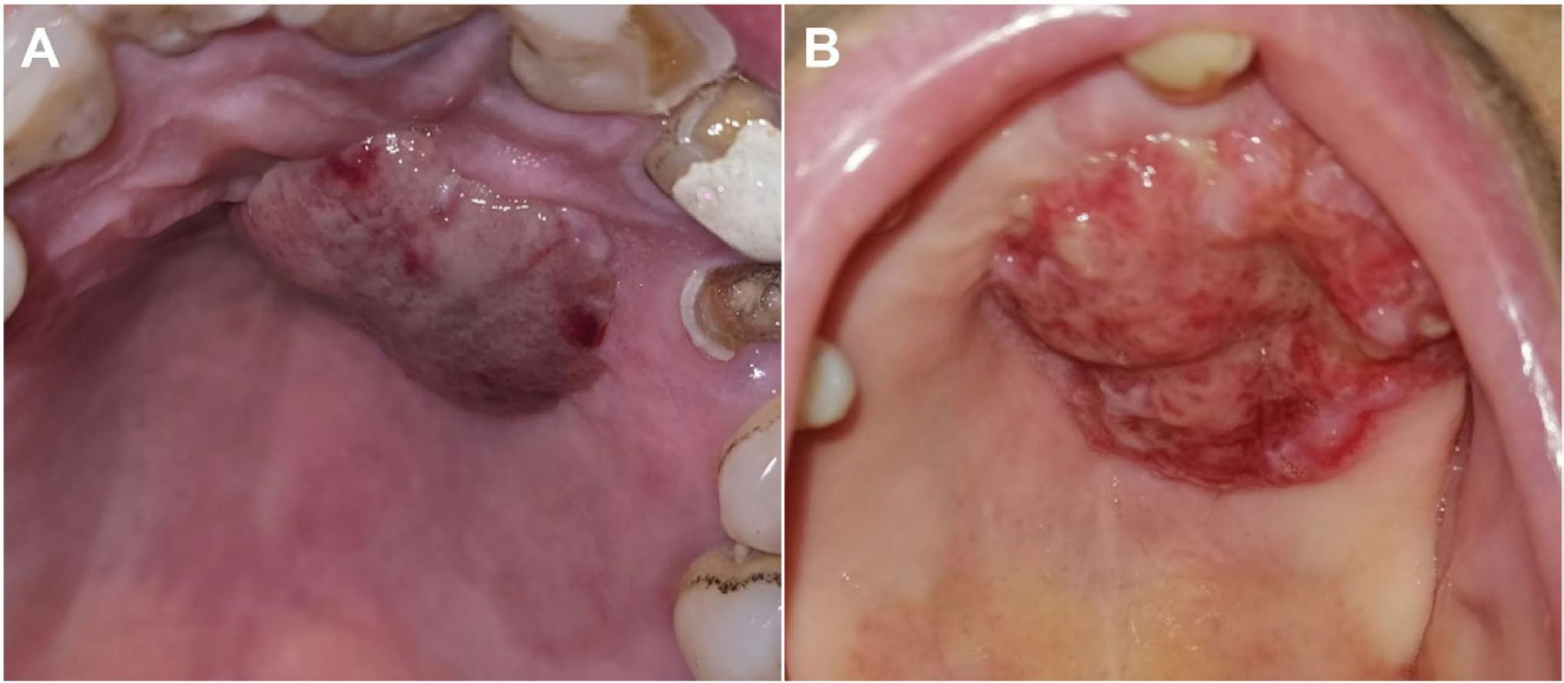
Clinical presentations of oral amelanotic melanoma cases. **A** Exophytic, sessile tumor involving the anteroposterior hard palate and adjacent palatal alveolar mucosa, exhibiting a predominantly pink to pale erythematous surface, irregular pattern, and focal hemorrhagic areas, without clinically evident pigmentation. **B** Exophytic tumor of the anteroposterior hard palate, with a lobulated contour, heterogeneous erythematous to reddish coloration, and extensive surface ulceration covered by fibrinous exudate, lacking visible melanin pigmentation

Most individuals were asymptomatic at diagnosis (*n* = 18; 78.3%), meaning that no symptoms (e.g., pain, bleeding, or obstruction) were reported, although clinically visible oral lesions were present (Fig. 1C). In 14 (66.7%) cases, the lesion was unifocal, whereas seven (33.3%) cases were multifocal (Fig. 1D). The palate/gingiva was the most frequently affected site (*n* = 16; 66.7%) (Fig. 1E**)**. Clinically, lesions most commonly presented as tumors (solid elevated lesions ≥ 2.0 cm in diameter) (*n* = 11; 37.9%) or macules (*n* = 8; 27.6%). Representative clinical features of melanotic and amelanotic oral melanomas are illustrated in Figs. 2 and 3, respectively. The mean lesion size was 3.8 ± 1.6 cm (range: 2.0–8.0 cm). Lesions measuring ≤ 3.5 cm accounted for 11 (53.4%) cases, whereas 10 (47.6%) cases measured > 3.5 cm (Fig. 1F). Regarding color, lesions were predominantly brown (*n* = 11; 52.4%) (Fig. 1H). Peripheral satellite pigmented lesions (“satelitosis”), characterized by small, non-elevated melanotic macules of varying shades of brown interspersed with areas of clinically normal mucosa, were a common finding. Clinically, this pattern was considered to be associated with the main melanoma and may assist in differentiating oral melanoma from other pigmented oral lesions (Fig. 2A, B, E, F, H, J). The most frequent clinical diagnostic hypothesis was oral melanoma (*n* = 15; 65.2%).

### Histopathological Features and Immunophenotypic Characterization

Melanotic melanoma was the most frequent histological subtype, observed in 16 (76.2%) cases, followed by amelanotic melanoma in three (14.3%) cases and melanoma in situ in two (9.5%) cases. Regarding cellular morphology, spindle-cell and mixed epithelioid/spindle-cell patterns were equally represented (*n* = 7 each).

**Fig. 4:**
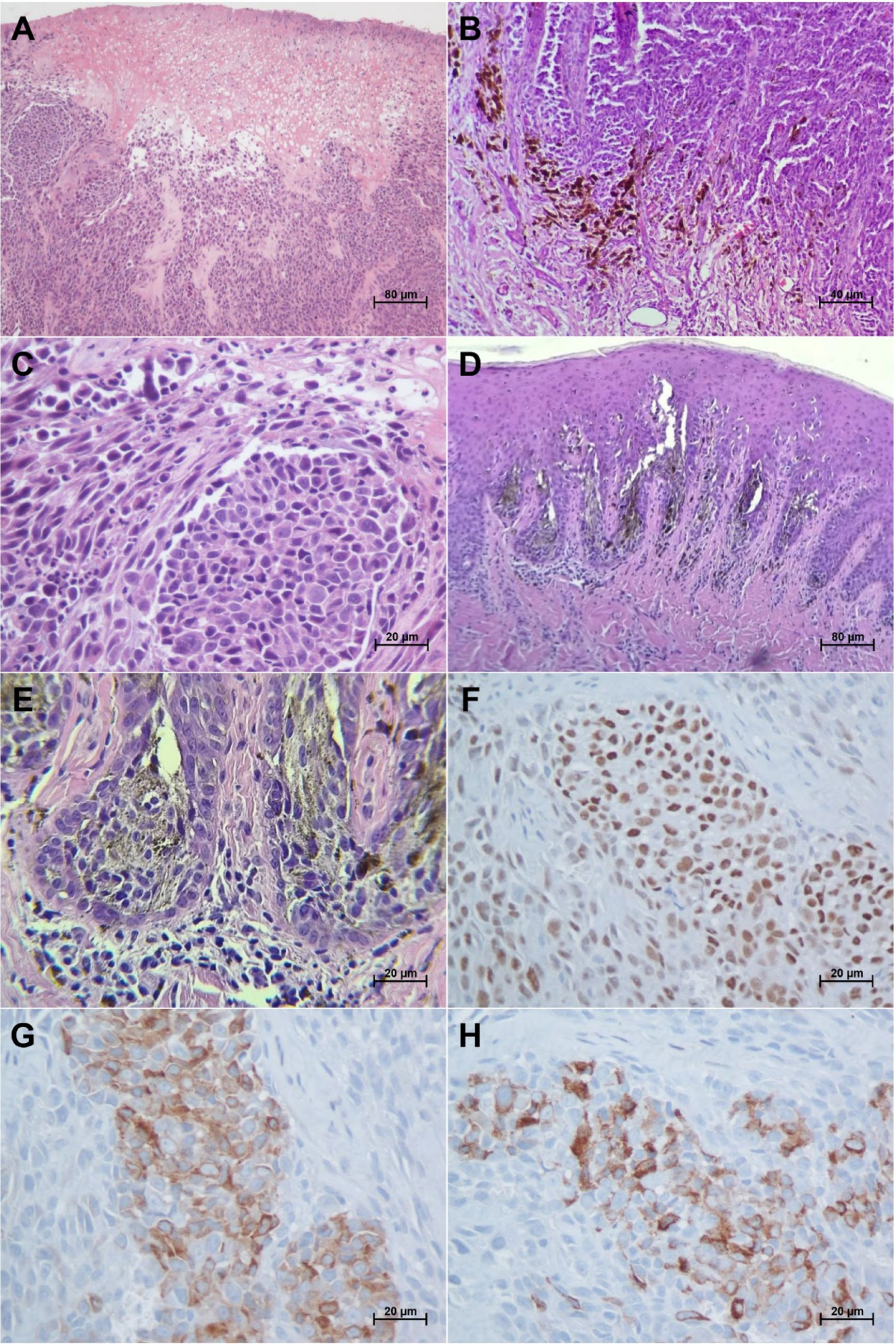
Histopathological and immunohistochemical features of oral melanotic melanoma. **A** Low-power view showing an invasive malignant melanocytic neoplasm infiltrating the lamina propria beneath the oral epithelium. **B** Sheets and nests of atypical melanocytic cells separated by delicate fibrous stroma, with conspicuous intracytoplasmic melanin pigment. **C** High-power view revealing marked cytologic atypia, including pleomorphic epithelioid and spindle-shaped cells, enlarged hyperchromatic nuclei, and prominent nucleoli. **D**, **E** Representative intraepithelial component/radial growth phase, showing atypical melanocytic proliferation along the epithelial junction with pigmentation. **F–H** Immunohistochemical staining showing **F** SOX10 with strong and diffuse nuclear positivity in tumor cells, **G** Melan-A with diffuse cytoplasmic positivity in tumor cells, and **H** HMB-45 with granular cytoplasmic positivity (hematoxylin and eosin staining, original magnifications: ×10, ×20, and ×40; immunohistochemistry, original magnification: ×40)

Immunohistochemical data were available for 15 of the 21 cases (71.4%). Among the tested cases, Melan-A expression was observed in all cases in which the antibody was applied (14/14; 100%), as were HMB-45 (13/13; 100%), SOX-10 (8/8; 100%), and S-100 protein (7/7; 100%). Cytokeratin AE1/AE3 expression was positive in 2/2 cases (100%). Proliferative activity assessed by Ki-67 was reported in two cases, with high labeling indices of 70% and 80% (Supplementary Table 2). Representative histopathological and immunophenotypic features of oral melanoma are illustrated in Fig. 4.

### Treatment and Outcomes

Surgical resection was performed in 18 (85.7%) individuals, either as a single modality or in combination with adjuvant therapies. Chemotherapy was administered in five (23.8%) individuals, and immunotherapy and/or radiotherapy were administered in selected patients (Fig. 1J). Regarding outcomes, nine (47.4%) individuals were alive, while 10 (52.6%) had died at the last follow-up. One patient developed distant metastasis involving the leg. Follow-up duration was available for nine patients, with a median of 12 months (range: 2–84 months). Two cases lacked follow-up information (Fig. 1K; Supplementary Table 1).

## Discussion

We herein contribute to the literature by documenting 21 additional cases of primary oral melanoma diagnosed in South America, thereby providing updated clinicopathological data from a region that remains underrepresented in large international studies [24]. The clinicodemographic and pathological features observed in our series are consistent with those described elsewhere [3–5]. According to the most comprehensive Latin American synthesis hitherto, Mexico accounts for approximately 40% of all reported cases of primary oral and sinonasal mucosal melanomas, followed by Brazil and Argentina, with additional contributions from Chile, Peru, Venezuela, Uruguay, and Paraguay. Of note, Colombia did not contribute identifiable oral melanoma series in that systematic review [3].

Most individuals in the present study were asymptomatic at diagnosis, reinforcing the well-established notion that oral melanoma frequently follows a clinically silent course in its early stages [4, 6]. Moreover, the age distribution observed in this series is consistent with recent literature indicating that oral melanoma predominantly affects older adults [2]. Although the mean age at diagnosis is commonly reported within the 65-69-year range [20], peak incidence during the seventh and eighth decades of life has also been recorded [25]. In the United States, for instance, the mean age at diagnosis is approximately 66.7 years, with nearly half of cases occurring in individuals older than 70 years [8, 26]. In contrast, South American studies tend to report slightly lower mean ages, averaging around 61 years [4], with a Brazilian series showing a mean age of 46 years [27]. Herein, two age peaks were identified, at 50–59 and 70–79 years, further underscoring the relevance of oral melanoma in older adults, particularly given the well-documented association between advanced age and poorer prognosis [2, 6, 20].

Sex distribution in oral melanoma remains heterogeneous across studies. Some authors report a male predominance [9, 21, 27], particularly in Asia and North America [20]. In our study, however, a slight female predominance was noted. This finding concurs with other studies reporting higher frequencies among women [8, 26] or no significant sex-based differences [4]. Clinically, oral melanomas typically exhibit asymmetrical and irregular patterns, presenting as nodular, tumoral, exophytic, macular, or ulcerated growths, with a wide spectrum of coloration ranging from gray to black or from red to purple, as also observed in the present study [2, 13]. Importantly, 10–30% of cases are amelanotic, which substantially complicates clinical recognition. Owing to these diagnostic challenges, amelanotic melanomas are associated with significantly poorer outcomes [1, 13]. In approximately 40% of affected individuals, mucosal hyperpigmentation precedes lesion development and may persist for months or years before malignant transformation [2, 28]; in the present study, one patient exhibited a previously unrecognized melanotic pigmentation.

Lesions predominantly involved the palate and maxillary gingiva. These sites are consistently identified as the most common anatomical locations for oral melanoma, accounting for 47.5–73.3% of cases [6, 20]. Although melanocytes are present in the basal layer of the oral epithelium and their density may vary across mucosal sites [29], current evidence does not clearly establish increased melanocyte density as the biological basis for this anatomical distribution [8]. Rambhia et al. [30] proposed that mechanical stress in masticatory mucosa may partly explain the preferential involvement of these sites in primary oral melanoma. Nevertheless, the mechanisms underlying the predilection for the palate and maxillary gingiva remain incompletely understood [8]. Lesion size has been reported to range from 0.8 to 12.0 cm, with a mean of approximately 4.5 cm [31]. Accordingly, advanced local disease at presentation remains common, as reflected by lesions exceeding 3.5 cm in nearly half of the cases in the current study. From a prognostic perspective, tumor size greater than 4 cm has been associated with poorer outcomes in oral melanoma [10]. Conversely, Boffano et al. [32] assessed depth of invasion but did not find a significant survival difference according to this parameter; therefore, its prognostic value in oral melanoma remains to be defined.

Diagnostic heterogeneity remains a central challenge in oral melanoma. As noted earlier, melanotic melanoma predominated in the current study; nevertheless, a clinically relevant proportion of cases was amelanotic and frequently associated with misleading initial diagnostic hypotheses, including non-neoplastic lesions (e.g., pyogenic granuloma). This observation is concordant with prior reports highlighting the propensity of amelanotic oral melanoma to mimic such conditions, thereby increasing the risk of misdiagnosis and inappropriate initial management [13, 14]. Pigmented oral lesions may also pose substantial diagnostic difficulty [5, 27]. The differential diagnosis of oral melanoma encompasses a wide array of solitary and multifocal pigmented conditions (e.g., amalgam tattoo, melanotic macule, melanoacanthoma, and acquired melanocytic nevus) [2, 27]. In this sense, the broad and heterogeneous diagnostic landscape underscores why pigmentation alone should not be regarded as a reliable discriminator and reinforces the need for a low threshold for biopsy of atypical, enlarging, ulcerated, or vascular-appearing oral lesions, regardless of color [2, 33]. Noteworthy, the “AEIOU” system has been proposed as a clinical aid for oral melanoma, defining “A” as age over 50 years, “E” as ethnicity (with higher prevalence among Asians, Hispanics, and Africans), “I” as irregularity of borders or color, “O” as the palate (the most common site), and “U” as ulceration [34]. Its applicability, however, appears to be limited to selected cases; thus, further studies are required to validate its diagnostic accuracy in routine clinical practice.

Histopathological examination supported by immunohistochemistry remains indispensable for the accurate diagnosis of oral melanoma. This principle is strongly reinforced by the current WHO classification of melanocytic tumors, which recognizes histopathology as the diagnostic gold standard, complemented, but not replaced, by ancillary techniques [23, 35]. In the present study, immunohistochemical analysis served as an important ancillary diagnostic tool, particularly in cases exhibiting spindle-cell, desmoplastic, or amelanotic features [11, 17]. This is relevant because oral melanoma may show microscopic overlap with poorly differentiated carcinomas, sarcomas, or lymphomas. Recognition of an intraepithelial component/radial growth phase, when present, is diagnostically relevant, as oral melanoma may show atypical junctional melanocytic proliferation adjacent to invasive disease [35]; however, this feature can be difficult to evaluate systematically in limited biopsy material. Saleem et al. [36] emphasized that no single immunohistochemical marker is sufficient in isolation, advocating instead a multimarker approach combining lineage-specific markers (e.g., Melan-A, HMB-45, SOX10, and S-100 protein), to improve diagnostic accuracy. In the oral mucosa, immunohistochemical interpretation requires caution because melanocytic markers differ in sensitivity, specificity, and performance across morphologic variants. SOX10 is particularly useful because of its nuclear staining pattern and high sensitivity, whereas S-100 protein is highly sensitive but less specific. Gazit and Daniels [37] showed that HMB-45 staining was stronger in round-cell oral melanomas, whereas S-100 predominated in spindle-cell melanomas. Melan-A is a melanocyte-specific cytoplasmic protein involved in the formation of stage II melanosomes and is therefore expected to be expressed in melanoma; nevertheless, its expression may be absent in a substantial proportion of cases, with reported positivity rates of approximately 70%. By contrast, HMB-45 has shown higher sensitivity, with reported positivity rates approaching 96% [17, 38, 39]. Together, these observations endorse the need for a multimarker, lineage-specific immunohistochemical approach.

Despite the limited and heterogeneous evidence defining the prognostic role of the Ki-67 labeling index in oral melanoma, available investigations suggest a potential association with clinical outcomes. In a cohort of 123 individuals with oral melanoma, Ma et al. [40] demonstrated that lesions exhibiting a Ki-67 index below 20% were more frequently associated with favorable survival outcomes. Similarly, an analysis of 175 patients with resectable mucosal melanoma indicated that Ki-67 expression may inform the need for adjuvant chemotherapy, with adjuvant treatment appearing particularly relevant in cases with Ki-67 indices of 30% or higher [41]. In oral melanoma, de Andrade et al. [36] reported Ki-67 indices ranging from 10.3 to 52.7%, whereas Soares et al. [42] found even higher indices in amelanotic oral melanomas, ranging from 34 to 92%, with a mean of 64%. Therefore, although the high Ki-67 labeling indices observed in selected cases in the present series align with the aggressive biological behavior of oral melanoma, the independent prognostic value of Ki-67 in mucosal melanomas remains incompletely defined and should be interpreted with caution [38].

Surgical resection remained the cornerstone of management and was performed in 85.7% of patients, either as a standalone approach or in combination with adjuvant modalities. This finding is consistent with current recommendations for head and neck mucosal melanoma, in which complete excision with negative margins represents the only potentially curative strategy [43, 44]. Although postoperative radiotherapy may improve local or locoregional control in selected cases, it has not demonstrated a clear survival benefit, reinforcing the concept that intensification of local therapy alone is insufficient to meaningfully alter the natural history of the disease [43, 45]. Recurrence remains a major concern in oral melanoma. In the present series, no local recurrence was observed, although one patient developed distant metastasis involving the leg. Literature has reported local recurrence rates ranging from 14 to 40% [4, 32], with no significant association between recurrence and histopathological variables (i.e., cell type, melanin, necrosis, vascular invasion, or perineural invasion) [4]. Regional nodal or distant metastases (e.g., lung, liver, brain, and bone) has been described in up to 62% of cases [4, 32].

Importantly, systemic strategies represent a critical future direction [46]. Mechanistically, oral melanoma should not be interpreted through a cutaneous melanoma paradigm alone. Mucosal melanomas generally lack a dominant ultraviolet mutational signature and show a lower point mutation burden than cutaneous melanoma, while displaying a higher burden of structural variants and copy-number alterations, including recurrent alterations involving *TERT*, *CDK4*, and *MDM2* [47]. These features may reduce neoantigen generation and partly contribute to the more modest responses to immune checkpoint inhibitors reported in mucosal melanoma compared with cutaneous melanoma [48, 49]. A recent systematic review indicated that the combination of radiotherapy with immune checkpoint inhibitors may enhance treatment efficacy in melanoma, including mucosal subtypes, by improving progression-free survival without a substantial increase in severe adverse events; nonetheless, no significant gains in overall survival at 6 or 12 months were documented [50]. In parallel, targeted therapies, including BRAF/MEK and KIT inhibitors, remain underexplored in oral melanoma, highlighting the need for molecularly driven therapeutic strategies and prospective studies tailored to this neoplasm [46, 48, 49].

Although poor outcomes in oral melanoma are often attributed primarily to intrinsic biological aggressiveness, compelling evidence supports a synergistic model in which aggressive tumor biology and delayed recognition interact to worsen prognosis [6, 10]. It is known that oral melanoma exhibits molecular characteristics distinct from those of its cutaneous counterparts and demonstrates a strong propensity for early systemic dissemination, even when local disease is adequately controlled [10, 12, 43]. The mortality observed in this study, i.e., approximately 53% at the last follow-up, closely parallels outcomes reported elsewhere, in which 5-year overall survival rarely exceeds 30–35% despite aggressive surgical management [6, 10]. Regarding amelanotic melanoma, the three instances documented here were alive at follow-up intervals ranging from three to 12 months. However, according to a systematic review by Bansal et al. [13], the estimated probability of 5-year survival is only 6.25%. The aggressive biological behavior of head and neck mucosal melanomas, including those arising in the oral cavity, is reflected in the staging system proposed in the American Joint Committee on Cancer 8th edition. In this system, mucosal melanomas are inherently classified as at least T3 disease at presentation. Thus, mucosal melanomas cannot be staged as T0, T1, or T2 [51].

Limitations of the present study warrant consideration. The retrospective design and modest sample size, both inherent to the rarity of oral melanoma, limited robust survival modeling and precluded analytical analyses. In addition, follow-up data were incomplete for a subset of cases, which may have led to an underestimation of late events. In some centers, once a diagnosis of oral melanoma was established, patients were referred to tertiary units for definitive treatment; consequently, long-term follow-up and detailed surgical information, including margin status, were not consistently available. Also, molecular assays were unfeasible at the participating centers. Conversely, the strengths of this study include the compilation of novel data on oral melanotic and amelanotic melanomas from five academic centers across three countries, enabling meaningful multicenter comparisons.

In summary, this multicenter South American series demonstrated that oral melanoma is an uncommon yet highly lethal malignancy, often asymptomatic at presentation and predominantly involving the palate and/or maxillary gingiva. Although surgery remains the mainstay of management, outcomes were poor, underscoring the need for heightened clinical suspicion and early biopsy of atypical oral lesions, including amelanotic presentations.

## Supplementary Information

Below is the link to the electronic supplementary material.

Supplementary Material 1

Supplementary Material 2

## Data Availability

No datasets were generated or analysed during the current study.
