# Supplementary material for: Oral Melanoma: A South American Collaborative Series of 21 Cases
Source: Head Neck Pathol. 2026 Jun 17;20(1):69. doi: 10.1007/s12105-026-01936-w (PMC13275972; doi:10.1007/s12105-026-01936-w)
Supplement: Supplementary file 1 — Supplementary Material 1 [file 12105_2026_1936_MOESM1_ESM.docx]

**Supplementary Table 1.** Data on clinicodemographic characteristics, treatment, and outcomes of oral melanoma cases from South America

| **Case** | **Age (years)** | **Sex** | **Skin color** | **Symptomatology** | **Anatomical location** | **Clinical aspects** | **Lesion size (cm)** | **Treatment** | **Follow-up** |
| --- | --- | --- | --- | --- | --- | --- | --- | --- | --- |
| **#1** | 51 | F | White | Asymptomatic | Palate | Red and brown tumor | 6.0 | S + CT + IT | Alive; 6 months |
| **#2** | 74 | M | White | Asymptomatic | Buccal mucosa/parotid region | Nodule | 2.0 | CT + IT | Alive; 18 months |
| **#3** | 54 | F | White | Asymptomatic | Mandibular gingiva | Nodule | 2.0 | CT + IT | Alive; 84 months; leg metastasis |
| **#4** | 86 | F | White | Asymptomatic | Palate | Ulcerated brown plaque | 3.0 | CT | Died; NR |
| **#5** | 76 | M | Black | Asymptomatic | Mandibular ridge/gingiva | Brown tumor | 4.0 | S | Died; NR |
| **#6** | 51 | F | Black | Asymptomatic | Maxillary gingiva | Brown tumor | 5.0 | S | Died; NR |
| **#7** | 80 | M | White | Weight loss and pain | Maxillary gingiva and lip | Ulcerated red and brown tumor | 8.0 | S | Died; NR |
| **#8** | 36 | M | Black | Asymptomatic | Palate and gingiva | Ulcerated tumor | 4.0 | S + CT | Died; NR |
| **#9** | 86 | F | NR | Asymptomatic | Maxillary gingiva | Pink exophytic tumor | 3.0 | S | Alive; 12 months |
| **#10** | 49 | F | NR | Asymptomatic | Palate | Black macule | 4.0 | S | Died |
| **#11** | 72 | M | NR | Asymptomatic | Maxillary gingiva | Brown tumor/macule | 4.0 | S | Died |
| **#12** | 75 | M | NR | Asymptomatic | Mandibular gingiva | Ulcerovegetative tumor | 2.0 | S | Alive; 5 months |
| **#13** | 30 | M | NR | Asymptomatic | Maxillary gingiva | Brown tumor/macule | 2.0 | S | Died |
| **#14** | 51 | M | NR | Asymptomatic | Palate | Black macule | 3.0 | S | Died |
| **#15** | 90 | F | NR | Asymptomatic | Palate and oropharynx | Black macule/tumor | 4.0 | S + RT | Died |
| **#16** | 51 | F | Non-White | Asymptomatic | Hard palate and gingiva | Black macule and reddish nodule | 3.0 | S | Alive; 2 months |
| **#17** | 62 | F | Non-White | Asymptomatic | Palate and maxillary gingiva | Brown and black macules with an ulcerated area | 5.0 | S | Alive; 36 months |
| **#18** | 76 | F | Non-White | Asymptomatic | Mandibular gingiva | Brown macules | 3.5 | S | Alive; 12 months |
| **#19** | 57 | M | White | Asymptomatic | Palate | Nodule | 2.0 | S | Alive; 3 months |
| **#20** | 60 | M | NR | Pain and obstruction of dental occlusion | Maxillary gingiva | Dark brown, granulomatous tumor | 7.0 | S | NR |
| **#21** | 56 | F | NR | Bleeding (history of intraoral pigmentation) | Palate and buccal mucosa | Dark-brown pigmentations | 3.0 | S | NR |

**Note:** CT, chemotherapy; F, female; IT, immunotherapy; M, male; NR, not reported; RT, radiotherapy; S, surgery.
