# Supplementary material for: Oral Melanoma: A South American Collaborative Series of 21 Cases
Source: Head Neck Pathol. 2026 Jun 17;20(1):69. doi: 10.1007/s12105-026-01936-w (PMC13275972; doi:10.1007/s12105-026-01936-w)
Supplement: Supplementary file 2 — Supplementary Material 2 [file 12105_2026_1936_MOESM2_ESM.docx]

**Supplementary Table 2.** Diagnostic hypotheses, histological subtypes, and immunohistochemical markers in oral melanoma cases from South America

| **Case** | **Clinical diagnostic hypothesis** | **Histological type** | **Immunohistochemical markers** |
| --- | --- | --- | --- |
| **#1** | Mucoepidermoid carcinoma and melanoma | Melanotic (epithelioid) | SOX-10++, HMB-45++, and Melan-A++ |
| **#2** | Melanoma | Melanotic (epithelioid) | SOX-10++, HMB-45++, Melan-A+, and S-100 protein++ |
| **#3** | Neoplasm | Melanotic (spindle cells) | HMB-45++, Melan-A+, and S-100 protein++ |
| **#4** | Melanoma | Melanotic (spindle cells) | HMB-45++, Melan-A++, and S-100 protein++ |
| **#5** | Melanoma | Melanotic (spindle cells) | HMB-45++, Melan-A++, and S-100 protein++ |
| **#6** | Melanoma | Melanotic (epithelioid/spindle cells) | HMB-45++, Melan-A++, and S-100 protein++ |
| **#7** | Melanoma | Melanotic (spindle cells) | HMB-45++, Melan-A++, and S-100 protein++ |
| **#8** | Neoplasm | Melanotic (spindle cells) | HMB45++ and S-100 protein++ |
| **#9** | Pyogenic granuloma | Amelanotic (epithelioid/spindle cells) | SOX-10++, HMB-45++, and Melan-A+ |
| **#10** | Melanoma | Melanoma *in situ* (epithelioid); radial/intraepithelial growth | Not performed |
| **#11** | Melanoma | Melanotic (epithelioid/spindle cells) | Not performed |
| **#12** | Neoplasm | Amelanotic (epithelioid/spindle cells) | SOX-10++, HMB-45++, and Melan-A++ |
| **#13** | Melanoma | Melanotic (desmoplastic) | Not performed |
| **#14** | Melanoma | Melanotic (epithelioid) | Not performed |
| **#15** | Melanoma | Melanotic (epithelioid) | Not performed |
| **#16** | Melanoma | Melanotic (epithelioid/spindle cells) | Not performed |
| **#17** | Melanoma | Melanotic (spindle cells) | SOX-10++ and Melan-A+ |
| **#18** | Reactive melanosis and melanoma | Melanoma *in situ* (epithelioid) ; radial/intraepithelial growth | Melan-A+ and AE1/AE3+ |
| **#19** | Pyogenic granuloma | Amelanotic (spindle cells) | SOX-10++, HMB-45++, Melan-A++, and AE1/AE3+ |
| **#20** | Squamous cell carcinoma | Melanotic (epithelioid/spindle cells) | SOX-10++, HMB-45++, Melan-A++, MIFT-1+, CK5/6-, p63-, E-cadherin-, p16-, enolase-, PGP9.5-, and Ki-67: 80%^1^ |
| **#21** | Melanoma | Melanotic (epithelioid/spindle cells) | SOX-10++, HMB-45++, Melan-A+, CK5/6-, and Ki-67: 70%^1^ |

**Note:** +, focal staining; ++, diffuse staining; -, negative staining.

^1^Ki-67 labeling index refers to nuclear staining in tumor cells, including those in the lamina propria and at the epithelial junction, when applicable.
